# 

**Published:** 1915-04-17

**Authors:** 


### Buildings Contemplated.

Crossgate Moor.—The Rural District Council have approved the plans for part of the scheme for the hospital which the Durham Guardians have decided to build at Crossgate Moor.

Evesham.—The Joint Hospital Board have decided to apply to the Local Government Board for sanction to borrow £1,000 for the enlargement of the administrative block at the hospital.

Great Driffield.—The East Riding of Yorkshire Hospital Committee invite tenders for enclosing and laying out the site and for the erection of various buildings in connection with a hospital for thirty-two beds. Quantities may be obtained of Mr. B. B. Stamford, architect, County Hall, Beverley.

Greenock.—The Greenock and District Combination Hospital Board are considering a proposal to extend the Gateside Hospital at an estimated cost of £1,800.

Ipswich.—The Local Government Board have held an inquiry into an application of the Town Council for permission to borrow £6,220 for the provision of a small-pox hospital at Foxhall Heath.

### Hospitals' Liability and Cases of Negligence.

It is well settled that the governors of a hospital are not liable for alleged negligence on the part of surgeons in performing an operation. The duty of the governors is to see that competent professional men are concerned with the hospital under their charge, but these surgeons are not the servants of the directors, so as to make the latter liable for any alleged negligence on their part. We notice that this principle also obtains in the American courts. In a recent case a charitable hospital was held liable for injuries to patients resulting from its negligence in not selecting proper servants and agents; thus while the particular decision was different, the general principle was the same. This, too, seems the rule relied on by the London County Council in a police case where a woman was charged with assaulting a County Council nurse on the ground that she had cut the hair of one of defendant's children at a County Council cleansing station. The judge expressed the opinion that the nurses of the London County Council should not take the extreme step of cutting a verminous school child's hair without a medical man's instructions. It was stated, on behalf of the County Council, that the nurses were specially trained for the work; that last year there were 9,000 cases dealt with, in about 70 per cent. of which the hair had to be cut, and the cutting was not done until at least six weeks' notice had been given to the patient. What would be the position of the County Council if injury to a child consequent on such an operation was alleged against it? Would it be enough for them to plead that they had employed sufficiently trained servants? Is the County Council nurse sufficiently qualified to say when a child's hair should be cut, or whether it was not practicable to clean the head without cutting the hair? On the whole, we incline to the opinion of the judge that in the case of older girls, at least, their hair should not be cut unless a doctor thought it necessary.

### Gifts and Bequests.

Mr. Alexander McOstrich, J.P., of Queenstown, County Cork, stockbroker and butter merchant, who left £346,403, of which £109,878 is in England, has bequeathed £100 each to the Victoria Hospital for Women and Children, Cork, and the Protestant Home for Incurables, Cork.

Miss Adelaide Slack, of West Didsbury, Manchester, has left estate of the gross value of £107,699. She bequeathed £200 each to the Manchester Royal Infirmary and to the Stockport General Infirmary.

Miss Elizabeth Mary Green, of Brighton, has left £1,000 to the Brompton Hospital for Consumption; £500 each to the Brighton, Hove, and Preston Dispensary, and the Brighton, Hove, and Sussex Throat and Ear Hospital; and after other legacies the residue of her property is left equally between the Cancer Hospital, Fulham, the Royal Hospital for Incurables, Putney, and the British Home for Incurables, Streatham.

### MEDICAL MISSION WORK.

The annual meeting of the Medical Missions Department of the Society for the Propagation of the Gospel in Foreign Parts is to be held in the Great Hall of the Church House at 8 p.m. on Wednesday, April 21. The chair will be taken by Sir Francis Champneys, Bart., M.D., and the speakers will be Mrs. Ferguson-Davie, M.D., of Singapore, and Dr. H. H. Weir, M.B., of Corea.

**Rates of Subscription**: Twelve months, 6/6; Six months, 4/-; Three months, 2/-, post free to any address in the United Kingdom. Abroad, Twelve months, 8/8; Six months, 5/-; Three months, 2/6, post free.—Address The Subscription Dept., "The Hospital," The Hospital Building, 28/29 Southampton Street, Strand, London, W.C.

### Publications.

**By Sir LAUDER BRUNTON, Bt., M.D., F.R.C.P., F.R.S., &c.**

SECOND EDITION. ALMOST ENTIRELY RE-WRITTEN.

### THERAPEUTICS OF THE CIRCULATION.

***Small Foolscap 8vo. With 111 Illustrations. Price 5s. net.***

"There can be few British medical men who are unacquainted with the author's lucid style and practical method of exposition. In this book they will find a sound and up-to-date account of the subject dealt with, and full descriptions of the various therapeutic measures employed in the relief of the symptoms of cardio-vascular disease. It may be cordially recommended to students and practitioners of medicine." *The Lancet.*

"The subject is dealt with by a master-hand."—*The Prescriber.*

**JOHN MURRAY, Albemarle Street, London.**

By SIR WILLIAM BENNETT, K.C.V.O., F.R.C.S.

***Crown 8vo. 5s. net.***

### INJURIES AND DISEASES OF THE KNEE JOINT

With 34 Illustrations.

NISBET & CO.

***Also, 8vo. 6s.***

FOURTH EDITION.

**Massage and Movements in Recent Fractures; Sprains and their Consequences; Rigidity of the Spine and the Management of Stiff Joints.**

With 23 Illustrations.

